# Prevalence, Clinical and Functional Determinants of Chronic Hypoxemia and Respiratory Failure in Patients with Stable COPD

**DOI:** 10.3390/jcm15124605

**Published:** 2026-06-13

**Authors:** Giacobbe Marco Giuseppe Ricco, Dejan Radovanovic, Matteo Pecchiari, Marina Saad, Juan Camilo Signorello, Francesca Mandurino Mirizzi, Michele Mondoni, Massimo Guerriero, Pierachille Santus

**Affiliations:** 1 Department of Biomedical and Clinical Sciences, Università degli Studi di Milano, 20157 Milano, Italy; giacobbe.ricco@unimi.it (G.M.G.R.); dejan.radovanovic@unimi.it (D.R.); 2 Division of Pulmonary Diseases, L. Sacco University Hospital, ASST Fatebenefratelli-Sacco, 20157 Milano, Italy; saad.marina@asst-fbf-sacco.it (M.S.); signorello.juan@asst-fbf-sacco.it (J.C.S.); mandurino.francesca@asst-fbf-sacco.it (F.M.M.); 3 Coordinated Research Center on Respiratory Failure, Università degli Studi di Milano, 20122 Milano, Italy; 4 Department of Pathophysiology and Transplantation, Università degli Studi di Milano, 20122 Milano, Italy; matteo.pecchiari@unimi.it; 5 Respiratory Unit, Department of Health Sciences, ASST Santi Paolo e Carlo, Università degli Studi di Milano, 20146 Milano, Italy; michele.mondoni@unimi.it; 6 Department of Computer Science, University of Verona, 37134 Verona, Italy; massimo.guerriero@univr.it

**Keywords:** hypoxemia, respiratory failure, cardiovascular comorbidities, COPD, lung function, air trapping

## Abstract

**Background and objective**: Hypoxemia and respiratory failure (RF) in chronic obstructive pulmonary disease (COPD) are associated with exacerbations, comorbidities and increased mortality. However, the prevalence of hypoxemia and RF in stable COPD is unknown. We aimed at investigating the prevalence and determining predictive factors for chronic gas exchange abnormalities in COPD patients. **Methods**: A retrospective cohort study that enrolled clinically stable COPD patients referring to a pulmonary outpatient clinic. Anthropometrics, clinical characteristics, blood gas analysis and lung function were analyzed. Patients were grouped according to hypoxemia (PaO_2_ <80 and ≥60 mmHg), type 1 (PaO_2_ < 60 mmHg) or type II (PaO_2_ < 60 and PaCO_2_ > 45 mmHg) RF. A sensitivity analysis adopting an age-adjusted definition of hypoxemia was performed. Predictive factors for hypoxemia or RF were assessed with multifactorial analysis. **Results**: We analyzed data from 515 patients. Fixed-ratio hypoxemia, RF type 1 and type 2 were observed in 352 (68.3%), 27 (5.2%) and 43 (8.3%) patients, respectively. Risk of hypoxemia was associated with preserved alveolar volume, residual volume/total lung capacity, and lung diffusion capacity. Heart failure, ischemic heart disease, atrial fibrillation, and metabolic syndrome were predictive factors for RF. Patients with age-adjusted hypoxemia (n = 321 patients, 62.3%) showed no difference in terms of anthropometrics, lung function, and clinical characteristics as compared with fixed-threshold hypoxemia. **Conclusions**: Hypoxemia is frequent in stable COPD. Lung function parameters and comorbidities can support the identification of patients at risk of RF. Blood gas analysis should be always performed in patients with COPD to allow for personalized therapy and management.

## 1. Introduction

Oxygen delivery and carbon dioxide removal are ensured by the functional coupling of the respiratory and cardiovascular systems [1]. Different pathophysiological alterations can decrease the oxygen tension in the arterial blood, thus causing hypoxemia, clinically defined as a partial pressure of oxygen in the arterial blood (PaO_2_) less than 80 mmHg [2]. As PaO_2_ is affected by age and altitude, hypoxemia can be more accurately defined by predictive equations, such as the one proposed by Sorbini and coworkers (i.e., PaO_2_ (mmHg) = 109 − 0.43 × (age in years), in the supine position, at sea level) [3].

When hypoxemia worsens and PaO_2_ drops below 60 mmHg in ambient air, respiratory failure (RF) occurs, defined as type I RF when carbon dioxide partial pressure (PaCO_2_) is within the physiological range, and type II RF when PaCO_2_ increases above 45 mmHg [4].

Hypoxemia and progressive RF represent a major complication in patients with chronic obstructive pulmonary disease (COPD), frequently associated with exacerbations leading to hospitalization [5,6,7], with a consequent high risk of mortality, ranging from 23% [8] to 80% [5,6,9,10]. A number of comorbidities are associated with hypoxemia in patients with COPD, including pulmonary vascular disease [11], skeletal muscle dysfunction [12], polycythemia [13] and heart failure [14]. Hypoxemia in COPD patients is caused mainly by ventilation/perfusion (V/Q) mismatch, reflecting inhomogeneity of regional ventilation secondary to alterations of the airways, or inhomogeneity of regional perfusion secondary to emphysema and damage of the lung capillary bed [5]. The V/Q mismatch associated with pulmonary emphysema or small airways disease is observable even in patients with mild COPD and worsens as the disease progresses [14].

The COPDGene study identified comorbid heart failure, pulmonary artery enlargement and severe COPD exacerbations as risk factors associated with the development of hypoxemia after a 5-year follow-up in patients with COPD [14]. However, oxygen saturation was measured by pulse oximetry, which may be inaccurate due to confounding factors and intrinsic limitations [15,16,17]. In fact, pulse oximetry consistently overestimates oxygen saturation in subjects with COPD [18], affecting treatment decisions, with low sensitivity and specificity observed especially in patients with a chronic bronchitis phenotype [18]. The gold standard for the assessment of gas exchange is arterial blood gas analysis (ABG) [19], which is invariably performed in acute conditions, but is still rarely available during stable disease [20,21,22]. Moreover, the clinical meaning of chronic hypoxemia in patients with COPD is still largely unknown.

The primary aim of this study was to investigate the prevalence of hypoxemia and RF, assessed with ABG, in clinically stable COPD patients. The secondary aim was to identify the predictive factors associated with hypoxemia or RF.

## 2. Materials and Methods

### 2.1. Study Design

A retrospective study was performed in a cohort of COPD patients attending the outpatient clinic of the Pulmonary Rehabilitation Unit of Fondazione Salvatore Maugeri Hospital, Milan (Italy), from December 2012 to January 2016.

### 2.2. Patients

Patients were consecutively enrolled according to the following inclusion criteria: (i) a confirmed diagnosis of COPD according to the lower limit of normal criteria [23]; (ii) stable clinical conditions—i.e., no exacerbations in the 8 weeks preceding enrolment; (iii) the ability to perform repeatable pulmonary function tests. Exclusion criteria included: impaired cognitive function estimated by a Mini-Mental State Examination score < 26, previous lobectomy, a restrictive and mixed obstructive-restrictive ventilatory pattern, and a previous history of asthma or other clinically significant respiratory diseases other than COPD. Patients who had recently undergone cardiothoracic surgery, with a New York Heart Association (NYHA) III or IV functional class, primitive pulmonary hypertension, diagnosis of vasculitis or other rheumatic diseases, and severe valvulopathies were also excluded. Patients were enrolled independently of the degree of airflow limitation or the presence of long-term oxygen therapy.

### 2.3. Functional Assessment

Arterial blood gas analysis (ABG) was performed at rest, by sampling the radial artery in room air conditions and analyzing the blood sample in the subsequent 30 s (GEM Premier 3000; Instrumentation Laboratory, Lexington, MA, USA). Patients on long-term oxygen therapy were put on ambient air conditions for 20 min before the ABG was performed. Static and dynamic lung volumes and total specific airway resistances (sRawtot) were assessed by means of a constant-volume body plethysmograph (MasterScreen Body; Erich Jaeger GmbH, Wurzburg, Germany). Intra-thoracic gas volume (ITGV) was measured at functional residual capacity, and sRawtot values were calculated during tidal breathing. The residual volume (RV) was obtained by subtracting the expiratory reserve volume from ITGV. The lung diffusion capacity for carbon dioxide (DLCO) was measured using the single breath technique (Master Screen PFT System; Jaeger, VIASYS Healthcare, Höchberg, Germany), in accordance with the European Respiratory Society (ERS)/American Thoracic Society (ATS) standards [24,25]. The CO transfer factor coefficient (KCO) was derived from the following equation: KCO = DLCO/VA, where VA is alveolar volume, assessed by using the inert gas dilution technique. Lung function testing was performed according to ATS/ERS recommendations [24]. For every patient, VA measurements were normalized to plethysmographic total lung capacity (TLC) [26,27,28]. The presence of ventilation inhomogeneity was considered as a VA/TLC < 0.8, as previously suggested [26,27]. Lung function and ABG were performed while patients were under the effect of chronic inhaled therapy, according to their specific long-term therapeutic regimen. Disease severity was graded according to the Global Initiative for Chronic Obstructive Lung Disease (GOLD) [6]. Comorbidities were retrieved from patients’ electronic forms. All measurements listed above, including height, weight, and body mass index, were assessed at the beginning of the study. The study was approved by the Central Ethics Committee (Fondazione Salvatore Maugeri-901 CEC) and conducted in accordance with the amended Declaration of Helsinki (2013), and each patient provided written, informed consent.

### 2.4. Study Groups

Patients were divided according to the presence of hypoxemia, defined as a PaO_2_ < 80 mmHg (fixed cutoff definition) [2,26]. Patients with normal PaO_2_ values (PaO_2_ ≥ 80 mmHg) belonged to group N (normal), while patients with hypoxemia formed group R (reduced). Group R was further stratified into patients with isolated hypoxemia (PaO_2_ ≥ and PaO_2_ < 80 mmHg, group H), with type I RF (PaO_2_ < 60 and PaCO_2_ ≤ 45 mmHg, group RF1), and type II RF (PaO_2_ < 60 and PaCO_2_ > 45 mmHg, group RF2). Group Hs included hypoxemic patients according to the definition of Sorbini and coworkers [2].

### 2.5. Study Outcomes

The primary outcome of the study was to investigate the prevalence of hypoxemia and RF, assessed with ABG, in clinically stable COPD patients.

The secondary outcome was to identify predictive factors associated with hypoxemia or RF (Type I or Type II) in patients with COPD.

A sensitivity analysis was carried out to compare the effects of defining hypoxemia using a fixed threshold (<80 mmHg) with the age-adjusted formula proposed by Sorbini and coworkers [2].

### 2.6. Statistics

Considering the paucity of data in the literature on the topic, the sample size could not be quantified. Quantitative data were expressed as mean and standard deviation (SD) or median and inter-quartile range (IQR), depending on their parametric distribution. Frequency and prevalence (percentage) were used to describe categorical variables.

The Shapiro–Wilk test was employed to test normality for continuous variables. The two-sample t-test or the Wilcoxon rank-sum test was used to compare the means of continuous variables for two independent groups. Chi-square test and the Exact Fisher test were used for analysis of the association between categorical variables. Multivariate logistic regression was used to study the predictive factors for the development of hypoxemia compared with type 1 and type 2 RF. The following independent variables were age, smoking habit, TLC, KCO, VA, VA/TLC, RV/TLC, number of comorbidities, heart failure, ischemic heart disease, liver disorder, atrial fibrillation and metabolic syndrome. Missing data for key variables (gas exchange, comorbidities, lung function) accounted for <3%; they were missing completely at random and therefore were not imputed. A *p*-value < 0.05 was considered statistically significant. Analyses were performed using STATA version 15 (StataCorp, College Station, TX, USA).

## 3. Results

### 3.1. Cohort Characteristics

**Figure 1:**
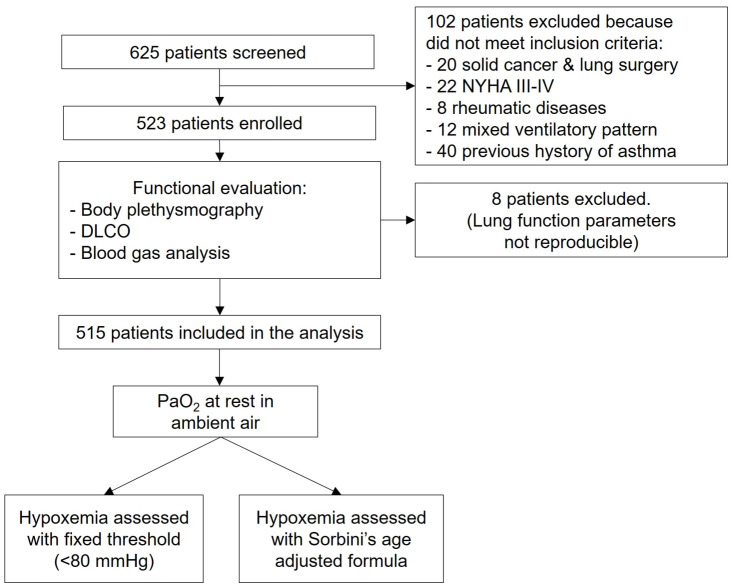
Study flowchart. DLCO: diffusion capacity of the lung for carbon dioxide; NYHA: New York Heart Association; PaO_2_: oxygen arterial pressure; PaCO_2_: carbon dioxide arterial pressure; RF: respiratory failure.

A total of 515 patients with stable COPD were enrolled, with a mean (SD) age of 73 (8.3) years, 30% females, with a predicted FEV_1_ of 51.5 (19.1%) (Figure 1, Table 1). On average, patients had an increased residual volume (RV) (163.6% predicted) and increased RV/TLC (141.8 (25.5%) predicted). DLCO was moderately reduced (47.1 (22.5%) predicted), with a more consistent reduction in KCO compared with VA (60% predicted vs. 79.7% predicted, respectively). Patients’ distribution according to GOLD category I, II, II and IV were 8.3%, 36.5%, 43.3%, and 11.8%, respectively. Fifty-one percent of patients had more than 1 moderate or severe exacerbations in the year before enrollment (Table 1). The most frequent comorbidities were hypertension (53.8%), metabolic syndrome (36.7%) and ischemic heart disease (28.2%). Data from ABG showed mean (SD) values of PaO_2_ and PaCO_2_ of 70.6 (10.2) mmHg and 44 (8.6) mmHg, respectively, and a pH of 7.43 (0.03) (Table 1). A complete list of patient functional and clinical characteristics is reported in Table 1.

### 3.2. Prevalence of Gas Exchange Abnormalities

**Figure 2:**
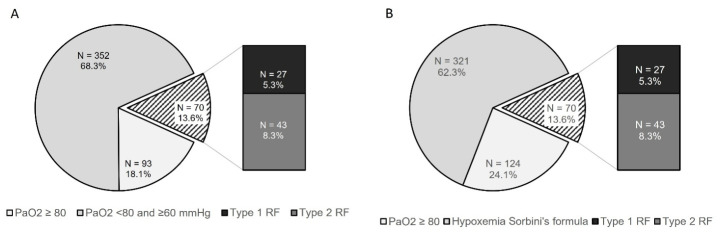
Patients’ distribution according to the severity of gas exchange abnormality. Panel (**A**) shows patients’ grouping according to the fixed PaO_2_ cutoff of 80 mmHg, while Panel (**B**) shows patients grouped when Sorbini’s age-adjusted formula was adopted to define hypoxemia. Note that the prevalence of patients with respiratory failure did not change from Panel (**A**) to Panel (**B**). RF = respiratory failure.

Within the study cohort, 93 (18.1%) patients had a PaO_2_ ≥ 80 mmHg (group N), and 422 (81.9%) patients had a PaO_2_ < 80 mmHg (Group R) (Figure 2A, Table 1). Of these, isolated hypoxemia according to the fixed threshold (PaO_2_ between 60 and 80 mmHg—Group H) was observed in 352 patients (68.3% of the study cohort), while when adopting Sorbini’s formula (age-adjusted hypoxemia—Group Hs), hypoxemia was observed in 321 patients (62.3% of the study cohort) (Figure 2A,B). Following the application of Sorbini’s formula, patients with a PaO_2_ ≥ 80 mmHg increased to 127 (24.1% of the study cohort). Twenty-seven patients (6.4% of the study cohort) had chronic type 1 respiratory failure (group RF1), and 43 patients (10.2% of the study cohort) had chronic type 2 respiratory failure (group RF2).

### 3.3. Normoxemic vs. Hypoxemic Patients

Anthropometrical characteristics, smoking history and comorbidities were comparable between groups (Table 1). As expected, gas exchange parameters were generally worse in hypoxemic compared with normoxemic patients. GOLD III and IV patients tended to be more prevalent in patients with hypoxia (45.7% vs. 32.3% and 13.0% vs. 6.4% in normoxemic and hypoxemic patients, respectively). Hypoxemic patients had generally worse airflow obstruction (FEV_1_ % predicted 58.6 vs. 50% predicted; *p* < 0.001 and FEV_1_/VC 74.5 vs. 64.3% predicted; *p* = 0.003), worse air trapping (RV 153 vs. 166.2% predicted; *p* = 0.041 and RV/TLC 136 vs. 143.2% predicted; *p* = 0.040) and worse diffusion capacity (DLCO 53.4 vs. 45.6% predicted, *p* = 0.006 and KCO 64.9 vs. 58.8% predicted, *p* = 0.037) as compared with normoxemic patients (Table 1).

### 3.4. Fixed Threshold vs. Sorbini’s Formula to Define Hypoxemia

Group H and Hs largely overlapped, with 314 patients meeting both hypoxemia thresholds, while only 38 patients were included in group H without being present in group Hs. Anthropometrical, clinical and lung function data were numerically very similar in all instances (Table 2 and Table 3). Considering the magnitude of the overlap between groups H and Hs, to avoid redundancy, a direct comparison between the two was not performed.

### 3.5. Type 1 vs. Type 2 Respiratory Failure

Active smoking and heart failure were statistically more prevalent in RF2 compared to RF1 patients (23.3 vs. 3.7%, *p* = 0.014 and 32.6 vs. 7.4%, *p* = 0.018, respectively). A significant association (*p* = 0.001) was found between the severity of airflow obstruction assessed with GOLD criteria and RF2 (Table 2). Patients in group RF2 had also significantly worse air trapping indexes (RV 138.4 vs. 191.8% predicted, *p* = 0.009; RV/TLC 126 vs. 162.6% predicted, *p* = 0.001), while DLCO and VA/TLC were comparable between groups (Table 3).

### 3.6. Predictive Factors for Hypoxemia and Respiratory Failure

A statistically significant association was observed in the likelihood to have hypoxemia rather than respiratory failure for the following lung function parameters: RV/TLC (OR 1.067, 95%CI: 1.017–1.118; *p* = 0.007), DLCO (OR 1.090, 95%CI: 1.015–1.170; *p* = 0.017) and VA (OR 1.266, 95%CI: 1.041–1.540; *p* = 0.018) (Table 4). For every increase in a % predicted unit of these parameters, there was a higher probability of having hypoxemia rather than RF.

Respiratory failure was independently predicted by active smoking (OR 0.252, 95%CI: 0.088–0.808; *p* = 0.020), heart failure (OR 0.019, 95%CI: 0.001–0.596; *p* = 0.024), ischemic heart disease (OR 0.077, 95%CI: 0.006–0.915; *p* = 0.042), atrial fibrillation (OR 0.090, 95%CI: 0.008–0.985; *p* = 0.049), metabolic syndrome (OR 0.042, 95%CI: 0.003–0.513; *p* = 0.013) and TLC (OR 0.897, 95%CI: 0.808–0.993; *p* = 0.038). The presence of cardiovascular comorbidities such as heart failure, ischemic heart disease, atrial fibrillation, and metabolic syndrome favored the presence of RF compared to the risk of hypoxemia (Table 4).

## 4. Discussion

Our study evaluated for the first time the prevalence of respiratory exchange alterations measured by ABG in a large population of clinically stable patients with COPD. Unlike other studies [14], which assessed the abnormality of gas exchange by means of pulse oximetry [18], the use of ABG also allowed for the unique differentiation of the patient subpopulation with PaO_2_ values < 80 mmHg in patients with chronic type 1 and type 2 respiratory failure, according to the levels of PaCO_2_. In a cohort of Norwegian COPD patients in stable clinical conditions, ABG was used to assess relationships between PaO_2_ and PaCO_2_ with clinical and functional variables [29], but the prevalence of gas exchange abnormalities was not investigated. According to the method of Sorbini and coworkers [3], we selected a group of patients (group Hs) that were almost indistinguishable from those of the hypoxemia group (H) evaluated by means of the fixed PaO_2_ threshold, confirming the possibility of using the fixed cutoff of 80 mmHg of PaO_2_ to define hypoxemia in clinical practice. In fact, a fixed cutoff could favor a rapid identification of patients with COPD with gas exchange abnormalities and an increased risk of adverse clinical outcomes, such as nocturnal hypoxemia, skeletal muscle dysfunction, cognitive impairment and vascular damage [5]. The fixed cutoff was also more sensitive than Sorbini’s formula for detecting hypoxemia, with a negligible increase in the risk of false positives.

The results of our study showed that an alteration of blood gases is a very frequent finding in patients with COPD in stable clinical conditions. Surprisingly, gas exchange abnormalities were present in 81.9% of patients, while chronic RF was present in 13.6%, in agreement with previous results [30], indicating that a significant reduction in PaO_2_ (on average patients had 69.6 ± 5.3 mmHg of PaO_2_) is not limited to acute exacerbations, but represents a common pathophysiological characteristic of COPD, even during clinical stability, suggesting the need of routine ABG monitoring, especially in patients with peculiar characteristics, such as more severe airflow obstruction and air trapping.

One unexpected result of the present study is the absence of patients with RF1 among patients with very severe airflow obstruction. Besides a sampling bias, this may be explained by the fact that GOLD IV patients usually have more pronounced hyperinflation and air trapping, and more significant regional ventilation heterogeneity [31]. As a consequence, they incur unfavorable mechanical coupling of the respiratory pump, leading to worse ventilation, with a predisposition to develop hypercapnia and therefore chronic type II respiratory failure. This is in line with a previous report showing that hypercapnia was associated with hypoxemia in patients with impaired ventilatory function, as in those assigned to GOLD IV [32], although this association was observed in COPD patients during an acute exacerbation and not in stable clinical conditions [32]. Indeed, a greater severity of the disease is neither necessary nor sufficient to detect serious alterations in ABG. Indeed, alterations in the V/Q ratio could already be observed in COPD patients with mild airflow obstruction [30,33]. However, these observations highlight the pathophysiological progression of respiratory failure, from milder disease characterized by V/Q mismatch and hypoxemia (type I RF) to advanced disease in which static and dynamic hyperinflation, increased dead space, and respiratory muscle dysfunction lead to increased blood PaCO_2_ and a higher risk of chronic type II RF.

A post-bronchodilator FEV_1_ cutoff of 36% predicted value was previously identified by other authors as the best threshold to identify RF1 and RF2 [34]. These findings are supported by a previous study, in which the level of PaO_2_ was correlated with the progression of COPD [35]. Accordingly, we found an average (SD) FEV_1_ of 39.8 (19.2%) predicted. We reason that, although spirometry alone cannot fully characterize gas exchange defects, FEV_1_ can be a practical indicator for the suspicion of gas exchange impairment and should consequently prompt further diagnostic assessments.

In our cohort, patients had, on average, two comorbidities, mostly cardiovascular, that allowed for a robust analysis of the potential association between comorbidities and gas exchange abnormalities. Nevertheless, cardio-metabolic comorbidities were generally not associated with hypoxia and/or respiratory failure, suggesting that clinical history alone is of limited informativeness in terms of blood gas alterations.

In contrast to other comorbidities, heart failure was significantly associated with RF2. This finding may be related to the greater degree of hyperinflation in subjects with RF2, which, according to previous studies, may negatively affect cardiac structure together with diastolic and systolic function [36,37,38]. In this context, a pharmacological reduction in hyperinflation may be able to partially reverse the constrictive cardiac pattern, improving cardiac mechanics [39,40,41].

Unique to our study was the assessment of predictive factors associated with respiratory failure. We showed that a higher TLC and a lower DLCO could be associated with a higher risk of respiratory failure, underlying the role of the alveolar-capillary barrier dysfunction and hyperinflation in the progression of gas exchange abnormalities in COPD. Moreover, we demonstrated that a positive history of heart failure, ischemic heart disease, atrial fibrillation, metabolic syndrome and active smoking were independently associated with a higher risk of respiratory failure than isolated hypoxemia. Indeed, heart failure and active smoking were shown to be associated with increased PaCO_2_ in a previous observational study [30]. We speculate that the presence of a high degree of hypoxemia and hypercapnia might be associated with autonomic disorders that may lead to a higher risk of atrial fibrillation. Moreover, atrial fibrillation, heart failure and ischemic heart disease might be responsible for an increase in V/Q mismatch due to interstitial edema, with reduced lung diffusion capacity. Indeed, the present study was not designed to investigate the pathophysiology of the underlying mechanisms of hypoxemia and RF, and a direct causal relationship between gas exchange abnormalities and lung function or comorbidities could not be inferred.

The present study suffers from several limitations. First, the study sample reflects the population accessing the outpatient clinic of a tertiary care academic rehabilitation center. Consequently, our cohort exhibits a higher disease severity and a greater burden of comorbidities compared to the general population of stable COPD patients managed in primary care, thereby limiting the generalizability of our observations, This specific clinical setting introduces a selection bias that may have led to an overestimation of chronic hypoxemia and gas exchange abnormalities, secondary to the high prevalence of patients with severe or very severe airflow obstruction. Second, we excluded patients with severe heart disease, which could have resulted in an underestimation of the true proportion of patients within the type 1 and type 2 respiratory failure groups. Finally, since the current study focused strictly on the point prevalence of gas exchange alterations, we did not evaluate longitudinal data to test the stability of arterial blood gas parameters over time.

In conclusion, our study showed that in patients with stable COPD, hypoxemia, irrespective of the definition applied, is a very frequent condition. Our data suggest that increased airway obstruction and air trapping, together with cardiovascular comorbidities, might represent predictive factors for chronic respiratory failure. Considering the clinical importance of chronic hypoxemia and respiratory failure in the context of COPD, ABG should be routinely performed not only in COPD patients during acute exacerbations, but also during clinical stability. Due to the retrospective nature of the study, causality between clinical, functional COPD characteristics and gas exchange abnormalities could only be hypothesized and stability of such findings over time could not be assessed. Prospective studies should further explore the role of timely identification and therapeutic optimization of gas exchange abnormalities, and their long-term association with important patient-related outcomes, such as symptoms, quality of life, exacerbations and mortality.

## Figures and Tables

**Table 1:** Demographic, clinical and functional parameters of the study population were divided by patients with (R) and without (N) hypoxemia. The definition of hypoxemia was PaO_2_ < 80 mmHg. B.E: base excess; BMI: body mass index; DLCO: lung diffusion capacity for carbon dioxide; FEV_1_: forced expiratory volume in first second; HCO^3−^: plasma bicarbonate; Kco: transfer factor coefficient for carbon monoxide; PaO_2_: arterial partial pressure of oxygen; PaCO_2_: arterial partial pressure for carbon dioxide; RV: residual volume; TLC: total lung capacity; VA: alveolar volume; VCMAX: slow vital capacity. Data are expressed as mean and standard deviation (SD) unless otherwise noted. Statistically significant differences (*p* < 0.05) are highlighted in bold. * Metabolic syndrome was defined as the presence of obesity (BMI ≥ 30 Kg/m^2^) variably associated with any of the following: arterial hypertension, dyslipidemia, hypercholesterolemia, diabetes or impaired glucose tolerance.

Variable	All Patients n = 515	N PaO_2_ ≥ 80 n = 93	R PaO_2_ < 80 n = 422	*p*-Value (N) vs. (R)
**Demographics**				
Males, n (%)	345 (70)	61 (65.6)	284 (67.3)	0.751
Age, years	73 (8.3)	71.7 (9.6)	73.3 (7.9)	0.469
BMI, Kg/m^2^	26.6 (5.9)	25.9 (4.9)	26.8 (6.1)	0.473
Smokers, n (%)	114 (22.1)	18 (19.3)	96 (22.7)	0.475
Smoke exposure, pack-years	58.8 (31.9)	52.5 (18.2)	59.8 (33.6)	0.471
**Disease status**				
Patients with ≥ 1 exacerbation, n (%)	263 (51.1)	44 (47.3)	219 (51.9)	0.436
Long-term oxygen therapy, n (%)	156 (30.3)	0 (0)	156 (36.9)	
GOLD I, n (%)	43 (8.3)	13 (14)	30 (7.1)	**0.003**
GOLD II, n (%)	188 (36.5)	44 (47.3)	144 (34.1)	
GOLD III, n (%)	223 (43.3)	30 (32.3)	193 (45.7)	
GOLD IV, n (%)	61 (11.8)	6 (6.4)	55 (13.0)	
**Comorbidities**				
Comorbidities per patient	2 (1.3)	1.9 (1.4)	2 (1.3)	0.473
Hypertension, n (%)	277 (53.8)	51 (54.8)	226 (53.5)	0.822
Metabolic syndrome *, n (%)	189 (36.7)	33 (35.5)	156 (36.9)	0.788
Ischemic heart disease, n (%)	145 (28.2)	23 (24.7)	122 (28.9)	0.417
Atrial fibrillation, n (%)	74 (14.4)	14 (15)	60 (14.2)	0.835
NYHA I-II heart failure, n (%)	69 (13.4)	8 (8.6)	61 (14.4)	0.134
Chronic liver disorder, n (%)	51 (9.9)	6 (6.4)	45 (10.7)	0.218
**Gas exchange parameters**				
pH	7.43 (0.03)	7.42 (0.04)	7.43 (0.03)	0.099
PaO_2_, mmHg	70.6 (10.2)	85.9 (6.9)	67.2 (7.3)	**<0.001**
PaCO_2_, mmHg	44.4 (8.6)	42 (6.3)	45.0 (8.9)	**<0.001**
HCO^3−^, mmol/L	29.6 (4.7)	28.3 (4.8)	30 (4.7)	**0.006**
B.E., mmol/L	4.9 (4.42)	3.4 (3.9)	5.3 (4.5)	**0.005**
**Lung function**				
DLCO, % predicted	47.1 (22.5)	53.4 (21.8)	45.6 (22.5)	**0.006**
K_CO_, % predicted	60 (27.0)	64.9 (23.2)	58.8 (27.8)	**0.037**
VA, % predicted	79.7 (14.8)	81.9 (16.0)	79.2 (14.5)	0.114
FEV_1_, liters	1.2 (0.5)	1.4 (0.6)	1.13 (0.5)	**<0.001**
FEV_1_, % predicted	51.5 (19.1)	58.6 (20.7)	50 (18.4)	**<0.001**
FEV_1_ < 50% predicted, n (%)	284 (55.7)	36 (39.8)	248 (58.2)	**0.001**
VC_MAX_, % predicted	80.3 (22.8)	84.3 (24.6)	79.4 (22.3)	0.073
FEV_1_/VC_MAX_	49.5 (17.3)	55.9 (20.3)	48 (16.1)	**0.002**
FEV_1_/VC_MAX_, % predicted	66.3 (23)	74.5 (27.0)	64.3 (21.5)	**0.003**
RV, % predicted	163.6 (56.7)	153 (53.2)	166.2 (57.3)	0.041
TLC, % predicted	111.0 (22.5)	108.7 (23.1)	111.6 (22.4)	0.187
V_A_/TLC	0.7 (0.1)	0.7 (0.1)	0.7 (0.1)	0.064
RV/TLC	60 (11.7)	57 (12.2)	60.7 (11.4)	**0.014**
RV/TLC, % predicted	141.8 (25.5)	136 (29.7)	143.2 (28.1)	**0.040**

**Table 2:** Anthropometric, clinical and gas exchange characteristics in patients with hypoxemia (fixed threshold—Group H), age-adjusted hypoxemia (Sorbini’s formula—Group Hs), type 1 chronic respiratory failure (group RF1), type 2 chronic respiratory failure (group RF2). B.E.: base excess; BMI: body mass index; HCO^3−^: plasma bicarbonate; PaO_2_: arterial partial pressure of oxygen; PaCO_2_: arterial partial pressure of carbon dioxide. Data are expressed as mean and standard deviation (SD) unless otherwise noted. Statistically significant differences (*p* < 0.05) are highlighted in bold. * Long-term oxygen therapy also includes patients with nocturnal hypoxemia on long-term nocturnal oxygen. ** Metabolic syndrome was defined as the presence of obesity (BMI ≥ 30 Kg/m^2^) variably associated with any of the following: arterial hypertension, dyslipidemia, hypercholesterolemia, diabetes or impaired glucose tolerance.

	H	Hs	RF1	RF2	
	60 ≤ PaO_2_ <80	Sorbini’s Formula	PaO_2_ < 60 PaCO_2_ ≤ 45	PaO_2_ < 60 PaCO_2_ > 45	*p*-Value (RF1 vs. RF2)
	n = 352	n = 321	n = 27	n = 43	
**Clinical variables**					
Males, n (%)	237 (67.3)	214 (66.7)	19 (70.4)	28 (65.1)	0.716
Age, years	73.7 (7.6)	72.8 (7.9)	71.2 (8.5)	71.2 (9.3)	0.991
BMI, Kg/m^2^	26.9 (6.2)	27 (6.3)	25.9 (3.9)	25.8 (6.5)	0.858
Smokers, n (%)	85 (24.1)	75 (23.4)	1 (3.7)	10 (23.3)	**0.014**
Smoke exposure, pack-years	60.5 (33.9)	62.8 (35.5)	17 (0.0)	58.1 (31.5)	0.203
**Disease status**					
≥1 exacerbation, n (%)	181 (51.4)	165 (51.4)	12 (44.4)	26 (60.5)	0.172
Long-term oxygen therapy, n (%)	86 (24.4) *	85 (26.5) *	27 (100)	43 (100)	**<0.001**
GOLD I, n (%)	24 (6.8)	21 (6.5)	4 (14.8)	2 (4.6)	**<0.001**
GOLD II, n (%)	128 (36.4)	118 (36.8)	11 (40.7)	5 (11.6)	
GOLD III, n (%)	163 (46.3)	144 (44.9)	12 (44.4)	18 (41.9)	
GOLD IV, n (%)	37 (10.5)	38 (11.8)	0 (0)	18 (41.9)	
**Gas analysis**					
pH	7.43 (0.03)	7.43 (0.03)	7.45 (0.03)	7.42 (0.05)	**0.001**
PaO_2_, mmHg	69.6 (5.3)	68.9 (5.2)	56.2 (3.3)	55 (3.9)	0.086
PaCO_2_, mmHg	44.1 (8.2)	44.3 (8.3)	38.8 (6)	55.6 (8.3)	**<0.0001**
HCO_3_^−^, mmol/L	29.5 (4.3)	29.6 (4.2)	27 (3.2)	35.9 (4.7)	**<0.0001**
B.E., mmol/L	5 (4.1)	5 (4)	2.5 (3.2)	10.5 (5)	**<0.0001**
**Comorbidity**					
Comorbidities per patient	2 (1.3)	1.9 (1.3)	2.2 (1.3)	2.2 (1.2)	0.450
Hypertension, n (%)	185 (52.6)	165 (51.4)	14 (51.8)	27 (62.8)	0.421
Metabolic syndrome, n (%) **	127 (36.1)	116 (36.1)	10 (37)	19 (44.2)	0.633
Ischemic heart disease, n (%)	104 (29.5)	90 (28)	8 (29.6)	10 (23.3)	0.656
Atrial fibrillation, n (%)	49 (13.9)	41 (12.8)	5 (18.5)	6 (13.9)	0.572
NYHA I-II heart failure, n (%)	45 (12.8)	40 (12.5)	2 (7.4)	14 (32.6)	**0.018**
Chronic liver disorder, n (%)	32 (9.1)	28 (8.7)	4 (14.8)	9 (20.9)	0.427

**Table 3:** Lung function parameters in patients with hypoxemia (fixed threshold—Group H), age-adjusted hypoxemia (Sorbini’s formula—Group Hs), type 1 chronic respiratory failure (group RF1), type 2 chronic respiratory failure (group RF2). DLCO: lung diffusion capacity for carbon dioxide; FEV_1_: forced expiratory volume in the first second; KCO: transfer factor coefficient for carbon monoxide; RV: residual volume; TLC: total lung capacity; VA: alveolar volume; VCMAX: slow vital capacity. Data are expressed as mean and standard deviation (SD) unless otherwise noted. Statistically significant differences (*p* < 0.05) are highlighted in bold.

	H	Hs	RF1	RF2	
	60 < PaO_2_ < 80	Sorbini’s Formula	PaO_2_ < 60 PaCO_2_ < 45	PaO_2_ < 60 PaCO_2_ > 45	*p*-Value (RF1 vs. RF2)
	n = 352	n = 321	n = 27	n = 43	
**Lung function**					
KCO, % predicted	61.4 (28)	60.9 (28)	43.7 (18.5)	44.9 (23.3)	0.479
V_A_, % predicted	79.7 (13.8)	80.2 (13.9)	76.6 (15.3)	76.3 (19.6)	0.433
DLCO, % predicted	47.9 (22.5)	47.8 (22.5)	33 (15.7)	33.1 (20.0)	0.421
FEV_1_, liters	1.1 (0.5)	1.2 (0.5)	1.4 (0.5)	0.9 (0.3)	**<0.001**
FEV_1_, % predicted	50.5 (17.9)	50.5 (18.4)	58.8 (16.7)	39.8 (19.3)	**<0.001**
FEV_1_ <50% predicted, n (%)	203 (57.7)	185 (57.6)	11 (40.7)	36 (83.7))	**<0.001**
VC_MAX_, % predicted	80 (21.8)	80 (22.4)	90.5 (27.8)	68.6 (21.2)	**0.047**
FEV_1_/VC_MAX_	48.2 (15.7)	48.7 (16.4)	53.9 (13.0)	43.2 (20.2)	**0.033**
FEV_1_/VC_MAX_, % predicted	64.6 (21)	65.2 (21.7)	71.7 (17.3)	57.5 (26.9)	**0.036**
RV, % predicted	164.8 (56.2)	167.3 (58.5)	138.4 (36.1)	191.8 (67.9)	**0.009**
TLC, % predicted	111.3 (21.9)	112.1 (22.3)	106.4 (21.4)	117.3 (26.4)	0.132
VA/TLC	0.7 (0.1)	0.7 (0.1)	0.7 (0.1)	0.6 (0.2)	0.213
RV/TLC	60.4 (11.3)	60.4 (11.8)	52.7 (10.5)	67 (10.5)	**<0.001**
RV/TLC, % predicted	142 (27.4)	143 (28.7)	126 (21.9)	162.6 (28.6)	**0.001**

**Table 4:** Multivariate logistic analysis for predictive factors for hypoxemia vs. type 1/type 2 respiratory failure. An odds ratio of > 1 is protective for RF, while an OR of < 1 is predictive of RF. Note that the 95% CI could appear skewed with respect to the OR due to the (ln)OR transformation. χ^2^ = 0.0006, R2 0.4178; significance threshold *p* < 0.05 in bold. For abbreviations, see Table 1 and Table 2.

Independent Variables	OR (CI, 95%)	*p*-Value
TLC	0.897 (0.808–0.993)	**0.038**
Age	1.106 (0.971–1.259)	0.127
V_A_	1.266 (1.041–1.540)	**0.018**
Smoking habit	0.252 (0.088–0.808)	**0.020**
Comorbidities (number)	2.560 (0.627–10.456)	0.190
NYHA I-II heart failure	0.019 (0.001–0.596)	**0.024**
Ischemic heart disease	0.077 (0.006–0.915)	**0.042**
RV/TLC	1.067 (1.017–1.118)	**0.007**
DLCO	1.090 (1.015–1.170)	**0.017**
Liver disorder	0.066 (0.004–1.085)	0.057
Atrial fibrillation	0.090 (0.008–0.985)	**0.049**
Metabolic syndrome	0.042 (0.003–0.513)	**0.013**
V_A_/TLC	5.09 × 10^−9^ (7.31 × 10^−18^ − 3.538)	0.066

## Data Availability

The database used for the current manuscript will be available from the Corresponding Author upon reasonable request.

## Abbreviations

The following abbreviations are used in this manuscript:

BMI	Body mass index
COPD	Chronic obstructive pulmonary disease
DLCO	Lung diffusion capacity for carbon monoxide
FEV_1_	Forced expiratory volume in 1 s
KCO	Transfer factor coefficient for carbon monoxide
PaO_2_	Partial pressure of oxygen in the arterial blood
PaCO_2_	Partial pressure of carbon dioxide in the arterial blood
RF	Respiratory failure
RV	Residual volume
TLC	Total lung capacity
VA	Alveolar volume
VCMAX	Slow vital capacity
